# The impact of non-TB mycobacterial co-isolation in patients with pulmonary TB

**DOI:** 10.5588/ijtldopen.25.0539

**Published:** 2026-03-13

**Authors:** S. Samari, S.K. Brode, H.K. Mah, M.S. Brar, N.F. Sabur

**Affiliations:** 1 Department of Medicine, McMaster University, Hamilton, ON, Canada;; 2 Division of Respirology, University Health Network, Toronto, ON, Canada;; 3 Department of Medicine, University of Toronto, Toronto, ON, Canada;; 4 Department of Surgery, University of Toronto, Toronto, ON, Canada;; 5 Division of Respirology, St. Michael’s Hospital, Toronto, ON, Canada;; 6 Li Ka Shing Knowledge Institute, Toronto, ON, Canada.

**Keywords:** sputum conversion, NTM pulmonary disease, pulmonary TB

## Abstract

**BACKGROUND:**

Pulmonary TB is diagnosed by isolating *Mycobacterium tuberculosis* from sputum. Culture conversion, defined as two consecutive negative sputum cultures, guides treatment duration. Non-TB mycobacteria (NTM) species are common in the environment but only cause pulmonary disease in certain patients, and the significance of co-isolation of NTM during TB treatment is unclear. We aimed to assess if NTM co-isolation impacts sputum conversion, treatment duration, or outcomes.

**METHODS:**

We conducted a retrospective study of pulmonary TB patients treated at West Park Healthcare Centre between 2010 and 2020. Demographics, microbiologic data, and clinical outcomes were extracted. Patients with NTM co-isolation were compared to those with TB-alone.

**RESULTS:**

Among 771 patients, 284 co-isolated NTM. These patients were older (median 58 vs. 49 years) and more often had diabetes (28.5% vs. 18.7%). They had longer time to sputum culture conversion (56 vs. 45.5 days) and were less likely to achieve sputum culture conversion (73.1% vs. 82.7%). NTM co-isolated patients had more persistent cough and sputum at treatment completion.

**CONCLUSION:**

NTM co-isolation in TB occurs more commonly in older patients, and those with diabetes. Patients who co-isolate NTM during TB treatment are less likely to culture convert, have a longer time to culture conversion, and have more end-of-treatment symptoms.

Diagnosis of pulmonary TB is based on sputum cultures demonstrating the presence of *Mycobacterium tuberculosis* (MTB). Treatment typically consists of antimicrobial therapy for 6 months, including a 2-month ‘intensive phase’ and a 4-month ‘continuation phase’. Routine sputum collection is performed until sputum culture conversion has been achieved, defined as two consecutive negative cultures at least 7 days apart following prior MTB positivity.^1^ Sputum collection in early phases of treatment is important, as sputum results contribute to decisions regarding de-isolation of patients, and culture conversion is a microbiologic benchmark that informs total treatment duration.^2^ Treatment duration is often extended to 9 months in the presence of persistent sputum culture positivity at the end of the intensive phase, as this signifies a higher burden of disease and greater risk of TB relapse.^3^

Non-TB mycobacterial (NTM) species are found in soil and water, and can cause significant pulmonary disease in certain patients. The clinical presentation and radiographic findings of NTM pulmonary disease (NTM-PD) are similar to those seen in pulmonary TB, though diagnosis requires exclusion of other processes, including pulmonary TB.^4^ Therefore, isolation of NTM in the sputum alone is not sufficient to make a diagnosis of NTM-PD in patients who have also cultured pulmonary TB. In Ontario, Canada, the prevalence of NTM-PD is high, estimated at 19 cases/100,000 in 2020.^5^ In patients with pulmonary TB in Ontario, NTM species have previously been isolated in the sputum of 11% of cases.^6^ Co-isolation of NTM in pulmonary TB poses a challenge to clinicians, as it can lead to persistent culture positivity, confounding decisions about de-isolation, and potentially leading to unnecessarily prolonged treatment. Additionally, the clinical significance of NTM co-isolation in pulmonary TB is unknown,^7–11^ and it is not clear if these patients are more likely to have persistent symptoms at the end of TB treatment, or if they have worse outcomes.^12^

We sought to determine whether co-isolation of NTM in patients with pulmonary TB resulted in differences in treatment duration, time to sputum culture conversion, end-of-treatment symptoms, or overall TB treatment outcomes.

## METHODS

West Park Healthcare Centre is a provincially designated TB treatment centre and is one of three adult TB clinics in the city of Toronto, servicing a population of approximately 3.03 million people, and a broader 6.5 million in the Greater Toronto Area.^13^ The estimated incidence of TB in Toronto is 10.7 cases per 100,000 population.^14^ We conducted a retrospective cohort study of all patients treated for TB at our institution between 1 January 2010 and 31 December 2020.

### Inclusion and exclusion criteria

We included patients who met the following criteria: age ≥18 years; diagnosis of pulmonary TB (by nucleic acid amplification testing [NAAT] or culture); and initiated TB treatment between 1 January 2010 and 31 December 2020. Patients were excluded from the study if they had a clinical diagnosis of TB disease (clinical presentation of TB but negative NAAT and/or culture); had extra-pulmonary disease with no pulmonary involvement; or had multidrug-resistant/rifampin-resistant TB.

### Data collection

Demographic data (including age and country of birth), microbiologic details (including smear and culture status), and clinical features (weight, disease location, comorbidities) at treatment initiation were retrospectively collected from patient charts. Additional data collected included number of sputa collected, time to smear/culture conversion, and clinical outcomes over a minimum 2-year follow-up period. End-of-treatment symptoms were abstracted as binary variables based on symptoms reported at the end of treatment and post-treatment completion visits.

### Definitions

Smear conversion was considered achieved when two consecutive sputum samples were found to be smear negative; culture conversion was achieved when two consecutive sputum samples were culture negative. The definition for culture conversion was modified slightly from the WHO, whereby sputum samples could be collected less than 7 days apart.^15^ Sputum collection was timed with clinical visits, and consecutive sputum samples were collected a minimum of one hour apart, in keeping with Canadian guidelines.^2^ The date of the first of two samples was deemed the date of smear/culture conversion. Patients who cultured an NTM organism on any sputum culture throughout the duration of treatment were considered to have co-isolation.

### Clinical outcomes

The primary outcome was total treatment duration. End-of-treatment outcomes followed 2013 WHO definitions: success (cure/treatment completion), treatment failure, death during treatment, and loss to follow-up/not evaluated.^15^ Cure was defined as a negative sputum smear or culture in the last month of TB treatment and on at least one previous occasion. The treatment failure definition was modified slightly from the WHO such that a regimen change due to adverse events alone was not considered a failure. All patients who had outcomes evaluated without loss to follow-up or death were evaluated for a minimum of 2 years post-treatment completion to assess for relapse.

### Statistical analysis

Descriptive statistics were calculated for baseline variables and microbiologic characteristics. Categorical variables were expressed as number (percentage) and mean (standard deviation) using the χ² or Fisher’s exact test; continuous variables were expressed as median (interquartile range [IQR]) using the Mann–Whitney *U* test. For binary treatment outcomes, exact binomial confidence intervals were reported. Statistical significance was set at *P* < 0.05. A multivariable logistic regression model was performed to assess whether age, BMI, and HIV status were associated with poor treatment outcomes. All analyses were performed using Stata v15 (Stata-Corp, College Station, TX, USA).

### Ethical statement

The study was approved by the West Park Healthcare Centre Research Ethics Board, Toronto, Canada.

## RESULTS

A total of 771 patients were included. Of these, 284 (36.8%) had co-isolation of MTB and an NTM species during treatment, while 487 (63.2%) cultured MTB alone (Table 1). The most common co-isolated NTM species were *M. avium*/*intracellulare* (77.4%), followed by *M*. *gordonae* (8.1%) and *M*. *abscessus* (3.2%) (Table 2). The median patient age was 52 years (IQR: 32–74), with NTM-TB patients significantly older than TB-only patients (58 [IQR: 37–78] vs. 49 [IQR: 30–71], *P* = 0.003). Women comprised 41% of the overall cohort, with no difference between groups (*P* = 0.72). The most common region of birth was Southeast Asia (64.7%), followed by the Americas (16.0%) and Africa (11.4%); this was similar between groups (*P* = 0.51). Patients with NTM-TB co-isolation were more likely to have diabetes mellitus than those with TB-only (28.5% vs. 18.7%, *P* = 0.002). The rates of HIV infection were similar between the two groups (2.5% vs. 2.1%, *P* = 0.71), as was the presence of smoking history (17.8% vs. 19.6%, *P* = 0.81).

**Table 1.** Baseline characteristics of NTM-TB and TB-only patients.

Characteristic	TB-only (n = 487)	NTM-TB (n = 284)	*P* value
Female sex, n (%)	202 (41.5%)	114 (40.1%)	0.72
Age (years), median (IQR)	49 (30–71)	58 (37–78)	0.003
Wt (kg), median (IQR)	57 (48–65)	57 (49–65)	0.24
Region of origin, n (%)			
Africa	57 (11.7%)	31 (11.0%)	0.51
Americas	82 (16.8%)	41 (14.5%)	
Eastern Mediterranean	11 (2.3%)	6 (2.1%)	
Europe	30 (6.2%)	12 (4.2%)	
Southeast Asia	307 (63.0%)	192 (67.8%)	
Western Pacific	0 (0%)	1 (0.4%)	
HIV infection, n (%)	10 (2.1%)	7 (2.5%)	0.71
Diabetes mellitus, n (%)	91 (18.7%)	81 (28.5%)	0.002
Smoking, n (%)	92 (19.6%)	49 (17.8%)	0.81
TB susceptibility, n (%)			
Fully sensitive	388 (86.4%)	217 (81.9%)	0.16
INH-R	44 (9.8%)	28 (10.6%)	
PZA-R	4 (0.9%)	4 (1.5%)	
TB location, n (%)			
Pulmonary	328 (67.4%)	196 (69%)	0.63
Pulmonary + extra-pulmonary	159 (32.7%)	88 (31%)	
Cavitary disease, n (%)	175 (35.9%)	120 (42.3%)	0.08
Bilateral disease, n (%)	247 (51.0%)	154 (54.4%)	0.37

NTM-TB = non-TB mycobacteria TB; IQR = interquartile range; INH = isoniazid; PZA = pyrazinamide.

**Table 2.** NTM species co-isolated.

Species	n (%)
*M. avium/intracellulare*	220 (77.4%)
*M. gordonae*	23 (8.1%)
*M. abscessus*	9 (3.2%)
*M. chelonae*	7 (2.5%)
*M. fortuitum*	7 (2.5%)
*M. xenopi*	7 (2.5%)
*M. mucogenicum*	6 (2.1%)
*M. kansasii*	1 (0.4%)
*M. lentiflavum*	1 (0.4%)
*M. obuense*	1 (0.4%)
*M. scrofulaceum*	1 (0.4%)
*M. shimoidei*	1 (0.4%)

Most patients had fully susceptible TB, with no significant difference between NTM-TB and TB-only patients (81.9% vs. 86.4%, *P* = 0.16). Presence of extra-pulmonary disease (31% vs. 32.7%, *P* = 0.63), cavitary disease (42.3% vs. 35.9%, *P* = 0.08), and bilateral disease (54.4% vs. 51.0%, *P* = 0.37) was also similar between groups.

### Treatment details

Treatment details are outlined in Table 3. Over the course of treatment, those with NTM-TB co-isolation had significantly more sputum samples collected compared to TB-only patients (median 10, IQR: 7–14, vs. 7, IQR: 4–11, *P* < 0.001). Sputum smear conversion rates between the NTM-TB and TB-only groups were similar (84.5% vs. 79%, *P* = 0.12), as was median time to smear conversion (46 vs. 43.5 days, *P* = 0.32). However, NTM-TB patients were less likely to achieve sputum culture conversion (73.1% vs. 82.7%, *P* = 0.014) and had longer time to culture conversion (56 vs. 45.5 days, *P* = 0.001).

**Table 3.** Bacteriological details for NTM-TB and TB-only patients.

Bacteriological characteristic	TB-only (n = 487)	NTM-TB (n = 284)	*P* value
Number of sputa collected^A^	7 (4–11)	10 (7–14)	<0.001
Smear conversion			
Achieved	245 (79%)	169 (84.5%)	0.12
Not achieved	65 (21%)	31 (15.5%)	
Time to smear conversion (days)^A^	43.5 (21–81)	46 (28–81)	0.32
Culture conversion			
Achieved	387 (82.7%)	201 (73.1%)	0.014
Not achieved	81 (17.3%)	74 (26.9%)	
Time to culture conversion (days)^A^	45.5 (24–70)	56 (32–82)	0.001

NTM-TB = non-TB mycobacteria TB.

A Presented as median (interquartile range).

### Treatment duration and outcomes

Treatment outcomes are outlined in Table 4. The overall treatment duration between NTM-TB and TB-only patients was similar (9 vs. 9 months, *P* = 0.84). There was a significant difference in treatment outcomes between NTM-TB and TB-only patients, with a higher rate of successful treatment completion and a smaller proportion of deaths in the NTM-TB group compared to TB-only patients (75.7% vs. 68.2% and 1.4% vs. 6.4%; *P* = 0.002) during the follow-up period. Final treatment outcomes were not available for a significant number of patients in both groups (21.1% in NTM-TB, 24.6% in TB-only). The difference in treatment outcomes between groups persisted even after controlling for age, BMI, and HIV status.

**Table 4.** Treatment outcomes.

Outcome	TB-only (n = 487)	NTM-TB (n = 284)	*P* value
Treatment duration (months)^A^	9 (6–12)	9 (9–12)	0.84
Treatment outcomes			
Died	31 (6.4%)	4 (1.4%)	0.002
LTFU	1 (0.2%)	1 (0.4%)	
Not evaluated	120 (24.6%)	60 (21.1%)	
Treatment completed	332 (68.2%)	215 (75.7%)	
Treatment failed	3 (0.6%)	4 (1.4%)	
End-of-treatment cough	46/332 (13.9%)	52/218 (23.9%)	0.003
End-of-treatment sputum production	12/332 (3.6%)	17/218 (7.8%)	0.03
End-of-treatment dyspnoea	10/332 (3%)	8/218 (3.7%)	0.67

NTM-TB = non-TB mycobacteria TB; LTFU = loss to follow-up.

A Presented as median (interquartile range).

Patients with NTM-TB co-isolation were more likely to have persistent cough (23.9% vs. 13.9%, *P* = 0.003) and sputum production (7.8% vs. 3.6%, *P* = 0.03) at the end of treatment. End-of-treatment dyspnoea was similar between the NTM-TB and TB-only groups (3.7% vs. 3.0%, *P* = 0.67).

## DISCUSSION

In this retrospective cohort study from a tertiary TB treatment centre in Toronto, Canada, NTM co-isolation was common, occurring in nearly 40% of pulmonary TB cases. Patients with NTM-TB co-isolation were significantly older and more likely to have diabetes, and were less likely to achieve sputum culture conversion; those who did had a longer time to culture conversion. NTM-TB patients were more likely to experience persistent cough and sputum production after completing TB treatment. Despite these differences, overall treatment success rate was slightly higher among NTM-TB patients.

Our findings differ from a recent retrospective study from Shanghai, which reported higher mortality among patients with NTM-TB co-infection, particularly those over age 65.^16^ However, a key methodologic difference was that the Shanghai study included only those patients who were considered to have true co-infection (≥2 positive sputum cultures, or bronchoalveolar lavage sample), whereas our study included any patient who co-isolated NTM during TB treatment. TB care in Ontario is de-centralised, and our centre is a tertiary referral centre where more complex or comorbid cases are often referred to establish treatment; this could have contributed to higher mortality in the TB-only group. Likewise, we had a high rate of ‘not evaluated’ outcomes, as many patients are transferred back to their treating physician/community hospital once treatment is established, and final outcomes therefore unavailable.

Geographic variation of NTM species between Ontario and China could also account for differences in treatment outcomes. In the Shanghai cohort, the most common NTM species isolated was *M. abscessus*, a rapid-growing organism which is typically more difficult to treat.^17^ In our cohort, the most frequently isolated species was *M. avium* complex, comprised of *M. avium* and *M. intracellulare*, which are slow-growing species not always associated with clinical disease.^17^ Similar to our findings, the Shanghai study reported comparable rates of cavitary disease between TB-only and NTM-TB groups.^16^ Another retrospective study from Taiwan found similar mortality rates between those with NTM-TB co-infection compared to those with TB alone, but those with co-infection required longer treatment duration for TB, primarily due to poor clinical response to treatment.^12^ These studies highlight the heterogeneity of patients NTM-TB co-isolation, and the limited understanding of its clinical significance.

The rate of NTM-PD is rising,^18^ and in Ontario, a 2020 retrospective review of Public Health Ontario’s laboratory records estimated the prevalence for *M. avium* pulmonary infection to be 13 per 100,000 persons, a 2.5-fold increase from 2010 (5.3 per 100,000 persons).^5^ Concurrent with this rise in NTM prevalence, there has also been an increase in NTM-TB co-isolation, with nearly 40% of cases co-isolating NTM with TB in our cohort, compared to only 11% of patients in 2004.^6^ Diagnosis of NTM-PD in the setting of concurrent pulmonary TB is challenging, as the diagnosis of NTM-PD requires a combination of clinical, radiographic, and microbiologic criteria, and exclusion of alternative diagnoses including pulmonary TB.^17^ Since pulmonary TB and NTM-PD have overlapping symptoms and radiographic features, it is difficult to distinguish to what extent NTM co-isolation represents colonisation versus true infection. There are no established guidelines on how to approach treatment of NTM-PD in the setting of ongoing treatment for pulmonary TB, although it is increasingly being recognised in various settings.^9–11,19,20^ Drug susceptibility information for non-TB mycobacterial isolates was not available in this study, and while it is possible that TB therapy partially treated the true NTM co-infections in our cohort, macrolides were not included in any TB treatment regimens, making NTM treatment incomplete. Our finding of persistent respiratory symptoms at the end of TB treatment in patients with NTM co-isolation suggests that at least a proportion of this population represent true NTM-TB co-infection, and therefore may benefit from a more tailored management strategy. In the Shanghai cohort, patients with established ‘co-infection’ were treated with a combination of both first line TB medications and NTM-specific treatments.^16^ Guidance on how to identify true co-infection from colonisation would be helpful in many settings, and represents an area for further investigation.

Microbiologic treatment response in pulmonary TB is monitored using routine sputum testing, with duration of therapy often being extended in the setting of persistent sputum culture positivity due to a higher risk of relapse.^21^ Our study demonstrates that patients with NTM-TB co-isolation are less likely to achieve culture conversion; therefore, sputum surveillance may be a less useful tool to monitor microbiologic response in this subpopulation of patients. In areas where there are high rates of NTM-TB co-isolation, sputum culture conversion under the current definition appears to be an increasingly impractical marker of treatment response. An alternative definition of culture conversion, perhaps only requiring the absence of MTB growth, should be considered, at least in areas where NTM is highly prevalent; in this case, more sophisticated laboratory techniques may be needed to avoid NTM overgrowth of viable MTB isolates.

Rising rates of NTM underscores the importance of identifying other measures of TB treatment response. While chest radiography can be a useful marker of treatment response, this is less reliable in the setting of NTM, since radiographic changes suggestive of TB are overlapping with NTM-PD.^22^ Several biomarkers have been explored in TB treatment and may be worth evaluating in the setting of NTM-TB co-isolation. A recent systematic review identified four serum biomarkers (CRP, IL-6, IP-10, and tumour necrosis factor-a) which improved after 8 weeks of TB therapy; further work is required to validate these biomarkers as a measure of treatment response and to investigate whether these correlate with risk of relapse.^23^ More novel blood-based assays of host mRNA associated with MTB are also being developed and show promise as potential measures of treatment response, as are sputum-based molecular assays measuring host RNA from MTB.^24–26^ Performance of these novel tests in the setting of NTM-TB co-isolation has not been established and warrants further attention.

Our study has several strengths. We included a large and ethnically diverse cohort of TB patients with NTM co-isolation in a setting where NTM is highly prevalent, but TB incidence is low. A relatively large number of patients with NTM-TB co-isolation, greater than in prior studies, allowed us to more accurately detect differences between groups. The study period spanned 10 years and included microbiologically confirmed cases of TB, with comprehensive follow-up data and long-term outcomes. Additionally, we included all patients who isolated NTM at any point during TB treatment, providing a real-world perspective on the clinical relevance of NTM co-isolation, even when full microbiologic criteria of NTM-PD may not be met. Our study also has several important limitations. First, this was a retrospective cohort and therefore we encountered missing data; notably, there was a large proportion of ‘not evaluated’ treatment outcomes, related to patients being transferred back to the original treating clinician. Second, our study did not differentiate between those with recurrent NTM culture positivity or those with a single positive NTM culture. This distinction may be clinically relevant, and will require attention in future studies. Finally, our study was conducted in a single TB centre in Ontario, and findings may not be generalisable to regions with differing epidemiology of NTM species.

## CONCLUSION

Our study highlights that NTM-TB co-isolation is common among patients with pulmonary TB, particularly in those who are older or with concurrent diabetes. Co-isolated patients have a longer time to culture conversion, are less likely to achieve culture conversion, and are more likely to have persistent pulmonary symptoms at the end of treatment. NTM co-isolation poses a challenge in utilising sputum culture conversion as a parameter of treatment response. Further research is needed to better understand this patient population, both in overall treatment approach and in monitoring response to treatment.

## References

[1] Linh NN, World Health Organization treatment outcome definitions for tuberculosis: 2021 update. Eur Respir J. 2021;58(2):2100804. 34413124 10.1183/13993003.00804-2021

[2] Cooper R. Appendix B: de-isolation review and recommendations. Can J Respir Crit Care Sleep Med. 2022;6(S1):248-255.

[3] Khan A, Lack of weight gain and relapse risk in a large tuberculosis treatment trial. Am J Respir Crit Care Med. 2006;174(3):344-348. 16709935 10.1164/rccm.200511-1834OC

[4] Griffith DE, An official ATS/IDSA statement: diagnosis, treatment, and prevention of nontuberculous mycobacterial diseases. Am J Respir Crit Care Med. 2007;175(4):367-416. 17277290 10.1164/rccm.200604-571ST

[5] Marras TK, Pulmonary nontuberculous mycobacteria, Ontario, Canada, 2020. Emerg Infect Dis. 2023;29(7):1415-1419. 37347810 10.3201/eid2907.230216 PMC10310396

[6] Damaraju D, Isolation of non-tuberculous mycobacteria among patients with pulmonary tuberculosis in Ontario, Canada. Int J Tuberc Lung Dis. 2013;17(5):676-681. 23575335 10.5588/ijtld.12.0684

[7] Sarro YD, Simultaneous diagnosis of tuberculous and non-tuberculous mycobacterial diseases: time for a better patient management. Clin Microbiol Infect Dis. 2018;3(3). 10.15761/CMID.1000144 PMC6319944 30613797

[8] Kendall BA, Isolation of non-tuberculous mycobacteria from the sputum of patients with active tuberculosis. Int J Tuberc Lung Dis. 2010;14(5):654-656. 20392362

[9] Hoza AS, Increased isolation of nontuberculous mycobacteria among TB suspects in Northeastern, Tanzania: public health and diagnostic implications for control programmes. BMC Res Notes. 2016;9:109. 26887928 10.1186/s13104-016-1928-3 PMC4756402

[10] Karamat A, Isolation of non-tuberculous mycobacteria among tuberculosis patients, a study from a tertiary care hospital in Lahore, Pakistan. BMC Infect Dis. 2021;21(1):381. 33894767 10.1186/s12879-021-06086-8 PMC8070300

[11] Aliyu G, Prevalence of non-tuberculous mycobacterial infections among tuberculosis suspects in Nigeria. PLoS One. 2013;8(5):e63170. 23671669 10.1371/journal.pone.0063170 PMC3650061

[12] Huang CT, Clinical significance of isolation of nontuberculous mycobacteria in pulmonary tuberculosis patients. Respir Med. 2009;103(10):1484-1491. 19467850 10.1016/j.rmed.2009.04.017

[13] Toronto. City of Toronto: Toronto at a Glance. https://www.toronto.ca/city-government/data-research-maps/toronto-at-a-glance/ (Accessed 23 January 2025).

[14] PHO. Epidemiologic summary: tuberculosis in Ontario, 2024. https://www.publichealthontario.ca/-/media/Documents/T/24/tuberculosis-ontario-epi-summary-2019-2024.pdf?rev=f95ec9f58de1478ebfc4052d87e44cdf&sc_lang=en&hash=23582C4EE01181A76D7DF9119DAB3317.

[15] WHO. Definitions and reporting framework for tuberculosis - 2013 revision. Geneva: WHO, 2013.

[16] Fei ZT, Clinical characteristics and outcomes of tuberculosis and non-tuberculous mycobacteria infections: a population-based study of concurrent and sequential infections. Eur J Clin Microbiol Infect Dis. 2025;44(8):1879-1887. 40343627 10.1007/s10096-025-05151-3 PMC12321909

[17] Daley CL, Treatment of nontuberculous mycobacterial pulmonary disease: an official ATS/ERS/ESCMID/IDSA clinical practice guideline. Clin Infect Dis. 2020;71(4):905-913. 32797222 10.1093/cid/ciaa1125 PMC7768745

[18] Prevots DR, Marras TK. Epidemiology of human pulmonary infection with nontuberculous mycobacteria: a review. Clin Chest Med. 2015;36(1):13-34. 25676516 10.1016/j.ccm.2014.10.002 PMC4332564

[19] Tan Y, Nontuberculous mycobacterial pulmonary disease and associated risk factors in China: a prospective surveillance study. J Infect. 2021;83(1):46-53. 34048821 10.1016/j.jinf.2021.05.019

[20] Otchere ID, Isolation and characterization of nontuberculous mycobacteria from patients with pulmonary tuberculosis in Ghana. Int J Mycobacteriol. 2017;6(1):70-75. 28317808 10.4103/2212-5531.201895 PMC5378301

[21] Johnston JCR, Menzies D. Chapter 5: treatment of tuberculosis disease. Can J Respir Crit Care Sleep Med. 2022;24(Suppl 1):66-76.

[22] Gopalan N, Predictors of unfavorable responses to therapy in rifampicin-sensitive pulmonary tuberculosis using an integrated approach of radiological presentation and sputum mycobacterial burden. PLoS One. 2021;16(9):e0257647. 34543329 10.1371/journal.pone.0257647 PMC8452066

[23] Zimmer AJ, Biomarkers that correlate with active pulmonary tuberculosis treatment response: a systematic review and meta-analysis. J Clin Microbiol. 2022;60(2):e0185921. 34911364 10.1128/jcm.01859-21 PMC8849205

[24] Sweeney TE, Genome-wide expression for diagnosis of pulmonary tuberculosis: a multicohort analysis. Lancet Respir Med. 2016;4(3):213-224. 26907218 10.1016/S2213-2600(16)00048-5 PMC4838193

[25] Zhang P, A blood-based 3-gene signature score for therapeutic monitoring in patients with pulmonary tuberculosis. Tuberc (Edinb). 2024;147:102521. 10.1016/j.tube.2024.102521 38801793

[26] Zainabadi K, An optimized method for purifying, detecting and quantifying Mycobacterium tuberculosis RNA from sputum for monitoring treatment response in TB patients. Sci Rep. 2022;12(1):17382. 36253384 10.1038/s41598-022-19985-w PMC9574834

